# Supplementary material for: Clinical and Immunologic Efficacy of the Recombinant Adenovirus Type-5-Vectored (CanSino Bio) Vaccine in University Professors during the COVID-19 Delta Wave
Source: Vaccines (Basel). 2022 Apr 21;10(5):656. doi: 10.3390/vaccines10050656 (PMC9143224; doi:10.3390/vaccines10050656)
Supplement: Supplementary file 1 [file vaccines-10-00656-s001.zip › vaccines-1650999-supplementary.pdf]

After vaccination	CanSino<br>*n*= 72<br>*n* (%)	Pfizer/<br>BioNTech<br>*n*=152<br>*n* (%)	AstraZeneca/<br>Oxford<br>*n*= 167<br>*n* (%)	Sputnik V<br>*n*=2<br>*n* (%)	Janssen/J&J<br>*n*=0<br>*n* (%)	CoronoVac<br>*n*=58<br>*n* (%)	Moderna<br>*n*=4<br>*n* (%)
Primary breakthrough symptomatic infection							
Total	3 (4.16)	2 (1.31)	7 (4.19)	0 (0.00)	0 (0.00)	2 (3.44)	0 (0.00)
Outpatient	3 (4.16)	2 (1.31)	6 (3.59)	0 (0.00)	0 (0.00)	2 (3.44)	0 (0.00)
Hospitalization	0 (0.00)	0 (0.00)	1 (0.59)	0 (0.00)	0 (0.00)	0 (0.00)	0 (0.00)
Supplemental oxygen	0 (0.00)	1 (0.65)	1 (0.59)	0 (0.00)	0 (0.00)	0 (0.00)	0 (0.00)
Mechanical ventilation or Death	0 (0.00)	0 (0.00)	0 (0.00)	0 (0.00)	0 (0.00)	0 (0.00)	0 (0.00)
Breakthrough symptomatic re-infection from previously infected participants							
Total	0 (0.00)	0 (0.00)	1 (0.59)	0 (0.00)	0 (0.00)	0 (0.00)	0 (0.00)
Outpatient	0 (0.00)	0 (0.00)	1 (0.59)	0 (0.00)	0 (0.00)	0 (0.00)	0 (0.00)
Hospitalization, Supplemental oxygen, Mechanical ventilation or Death	0 (0.00)	0 (0.00)	0 (0.00)	0 (0.00)	0 (0.00)	0 (0.00)	0 (0.00)

Table S1. Clinical outcomes of patients presenting COVID-19 breakthrough infections and reinfections after partial vaccination.

Not vaccinated population	***n= 63 (%)***
Total	63 (100)
Outpatient	62 (98.41)
Hospitalization	1 (1.58)
Supplemental oxygen	2 (3.17)
Mechanical ventilation	0 (0.00)
Death	0 (0.00)

Table S2. Clinical outcomes of unvaccinated participants.

After vaccination	CanSino *n*= 5,432	Pfizer/ BioNTech *n*=771	AstraZeneca/ Oxford *n*= 633	Sputnik V *N*=7	Janssen/J&J *n*=58	CoronoVac *n*=155	Moderna *n*=94
Primary breakthrough symptomatic infection in fully vaccinated							
Total	224	14	10	0	2	1	2
Age, mean (SD)	40.7 (±9.5)	50 (±13.7)	64.8 (±7.1)	NA	42.5 (±9.1)	53	51.5 (±4.9)
Female (%)	107 (47.7)	8 (57.1)	2 (20.0)	0 (0.00)	1 (50.0)	1 (100.0)	2 (100.0)
Diabetes (%)	15 (6.6)	2 (14.2)	3 (30.0)	0 (0.00)	0 (0.00)	1 (100.0)	0 (0.00)
Hypertension (%)	19 (8.4)	3 (21.4)	1 (10.0)	0 (0.00)	0 (0.00)	0 (0.00)	0 (0.00)
Obesity (%)	37 (16.5)	2 (14.2)	2 (20.0)	0 (0.00)	0 (0.00)	1 (100.0)	0 (0.00)
Breakthrough symptomatic re-infection from previously infected fully vaccinated participants							
Total	15	1	2	0	0	0	0
Age, mean (SD)	37.7 (±9.8)	43	59.5 (±6.3)	NA	NA	NA	NA
Female (%)	7 (46.6)	1 (100.0)	1 (50.0)	0 (0.00)	0 (0.00)	0 (0.00)	0 (0.00)
Diabetes (%)	1 (6.6)	0 (0.00)	0 (0.00)	0 (0.00)	0 (0.00)	0 (0.00)	0 (0.00)
Hypertension (%)	0 (0.00)	0 (0.00)	1 (50.0)	0 (0.00)	0 (0.00)	0 (0.00)	0 (0.00)
Obesity (%)	4 (26.6)	0 (0.00)	0 (0.00)	0 (0.00)	0 (0.00)	0 (0.00)	0 (0.00)
Primary breakthrough symptomatic infection in partially vaccinated							
Total	3	2	7	0	0	2	0
Age, mean (SD)	45 (±7)	37.5 (±4.9)	48.1 (±6.8)	NA	NA	46 (±2.8)	NA
Female (%)	1 (33.3)	0 (0.00)	3 (42.8)	0 (0.00)	0 (0.00)	1 (50.0)	0 (0.00)
Diabetes (%)	0 (0.00)	0 (0.00)	2 (28.5)	0 (0.00)	0 (0.00)	0 (0.00)	0 (0.00)
Hypertension (%)	1 (33.3)	0 (0.00)	2 (28.5)	0 (0.00)	0 (0.00)	0 (0.00)	0 (0.00)
Obesity (%)	0 (0.00)	0 (0.00)	2 (28.5)	0 (0.00)	0 (0.00)	0 (0.00)	0 (0.00)
Breakthrough symptomatic re-infection from previously infected partially vaccinated participants							
Total	0	0	1	0	0	0	0
Age, mean (SD)	NA	NA	29	NA	NA	NA	NA
Female (%)	0 (0.00)	0 (0.00)	1 (100.0)	0 (0.00)	0 (0.00)	0 (0.00)	0 (0.00)
Diabetes (%)	0 (0.00)	0 (0.00)	0 (0.00)	0 (0.00)	0 (0.00)	0 (0.00)	0 (0.00)
Hypertension (%)	0 (0.00)	0 (0.00)	0 (0.00)	0 (0.00)	0 (0.00)	0 (0.00)	0 (0.00)
Obesity (%)	0 (0.00)	0 (0.00)	0 (0.00)	0 (0.00)	0 (0.00)	0 (0.00)	0 (0.00)

Table S3. Clinical characteristics of patients presenting COVID-19 breakthrough infections and reinfections according to gender and the most common comorbidities. NA: Not applicable.

	n= 352<br>*n (%)*	*S antibody value*<br>*mean (SD)*
Vaccine		
Cansino	311 (88.3)	6,043 ±17,266.8
Pfizer	20 (5.6)	4,036.1 ±7,586
AstraZeneca	21 (5.9)	244.9 ±188.1
Group		
Breakthrough infection	69 (19.6)	21,483.4 ± 30,28.1
Reinfection	3 (0.8)	3,373.1 ± 2,756.1
Previous infection	61 (17.3)	5,055 ± 6,835.3
Without infection	219 (62.2)	1,420.3 ± 4,700.6

Table S4. Number of participants serologically tested and mean S antibody production according to administered vaccine and self-reported COVID-19 infection.
